# Non-invasive Tear Film Assessment in Normal Population: Effect of Age, Sex, and Interparametric Relationship

**DOI:** 10.3389/fmed.2022.894184

**Published:** 2022-06-01

**Authors:** Swati Singh, Saumya Srivastav, Ashik Mohamed, Sayan Basu

**Affiliations:** ^1^Centre for Ocular Regeneration, L V Prasad Eye Institute, Hyderabad, India; ^2^Ophthalmic Plastic Surgery Services, L V Prasad Eye Institute, Hyderabad, India; ^3^Ophthalmic Biophysics Laboratory, L V Prasad Eye Institute, Hyderabad, India; ^4^The Cornea Institute, L V Prasad Eye Institute, Hyderabad, India

**Keywords:** non-invasive tear break up time, tear meniscus height, tear osmolarity, tear film parameters, meibography

## Abstract

**Purpose:**

To investigate age- and sex-related differences in tear film parameters of normal Indian population and study interparametric relationships.

**Methods:**

Healthy subjects with no ocular disease (median ocular surface disease index = 0) were subjected to an automated evaluation of tear meniscus height (TMH), non-invasive tear breakup time (NIBUT) using Keratograph 5M (OCULUS GmbH, Wetzlar, Germany), and tear osmolarity using the TearLab Osmolarity System (TearLab Corporation, California, USA). A mixed-effects model with random intercepts at the patient level was used to evaluate the relationships between explanatory (age, gender, and tear osmolarity) and outcome variables (TMH and NIBUT).

**Results:**

A total of 237 subjects (474 eyes; 150 males) were enrolled with a mean age of 40 ± 17 years (range, 10-78 years). The mean values (± standard deviation) of TMH, NIBUT, and tear osmolarity were 0.34 ± 0.07 mm, 10.95 ± 2.02 s and 289.0 ± 5.8 mOsm/L, respectively. Age had a significant positive relationship with TMH (*p* < 0.0001; 0.002 mm/year; r = 0.12), but there was no effect on NIBUT (*p* = 0.26) and tear osmolarity (*p* = 0.27). There were no sex-based differences in tear film parameters. Interparametric relationship revealed no significant association between TMH and NIBUT (*p* = 0.12) or tear osmolarity and TMH (*p* = 0.83) or tear osmolarity and NIBUT values (*p* = 0.48).

**Conclusions:**

In a normal Indian population, TMH is weakly affected by age and is independent of sex, NIBUT, and tear osmolarity. Tear breakup time and osmolarity show no significant age- and sex-related variation.

## Introduction

With the introduction of non-invasive diagnostic modalities, dry eye disease (DED) diagnosis relies on an objective assessment of tear film characteristics (1). The variation in tear film parameters according to age and sex needs to be understood as higher incidence of DED is reported in older individuals and more in women (2, 3). The age and sex-based differences in the prevalence of DED have been attributed to the age-related changes in tear film dynamics and lacrimal gland atrophy. However, these differences have not been uniformly observed across the published literature. Many studies have reported no effects of age on the tear film in normal individuals, whereas few have found it otherwise (4–14). There is still a lack of conclusive evidence explaining the dry eye symptomatology correlation with increasing age and female sex. The studies with proper methodology, where the effect of age and gender on tear physiology have been investigated in extensive population-based studies, are scarce and mainly from the European or American continent. However, normative data for these parameters have shown variation among different populations, and the values from one population where the machine has been developed are taken as a reference for making a DED diagnosis. Also, it is not easy to draw any comparison between existing studies as different instruments like tearscope, evaporimetry, and gamma scintigraphy for measuring tear stability, have been utilized across studies. With the introduction of non-invasive diagnostic machines that provide a comprehensive, objective assessment of tear film and lack subjective bias, we have tried to explore them to address age- and gender-related changes in the tear film. This study aims to investigate the age and sex-based differences in tear film parameters in a large cohort of the normal Indian population and study their interparametric relationship.

## Methods

### Study Subjects

This study followed the tenets of the Declaration of Helsinki and was approved by the Institutional Ethics Committee. This prospective study enrolled 237 healthy subjects belonging to 10-78 years of age (*N* = 40 per decade; except last decade) recruited from hospital staff volunteers. After obtaining informed consent, the ocular surface disease index (OSDI) questionnaire was filled for every participant. Criteria for labeling normal was OSDI < 13, no ocular symptoms (asked verbally), and no ocular staining (slit lamp examination). Excluded were the individuals who underwent ocular surgery, had lid abnormalities (such as ectropion, entropion), contact lens wearers, or taking any ocular /systemic medications known to affect the tear film, ocular injury, or other ocular diseases such as ocular infection, allergy, or any systemic autoimmune disease.

### Automated Tear Film Parameters Measurements

The tear film was assessed using the Oculus Keratograph 5M (OCULUS GmbH, Wetzlar, Germany) and TearLab Osmolarity System (TearLab Corporation, California, USA). The controlled environment chamber (CAE internal dimension of 6′ × 5′ × 8′, temp range of 25 ± 1.0°C, humidity range of 44 ± 5.0%, and display LCD 200 lux) was used for maintaining similar environmental conditions. The measurements were taken by the single observer (S.S.) following the TFOS DEWS II Diagnostic Methodology article report on the same day between 10:00 and 16:00. The assessment was carried out in the following order: Ocular surface disease index questionnaire (OSDI), tear osmolarity, tear meniscus height (TMH), and non-invasive tear breakup time (NIBUT). There was randomization of the eye to be tested first and a 5-min interval was kept between every measurement. The other eye was tested 20 s (s) after the first while evaluating NIBUT.

### Statistical Analysis

Readings from both eyes of each individual were included in analysis. A mixed-effects model with random intercepts at the patient level was used to evaluate the relationships between age, gender, and tear osmolarity and outcome variables of TMH and NIBUT. Normality distribution for each group and the total sample was analyzed through Shapiro-Wilk test based on the sample size. Correlations between the tear film parameters and the age and gender were analyzed using the binomial logistic regression for predicting the tear film metrics to that of the statistically significant correlations. The *p*-value < 0.05 was considered statistically significant.

## Results

**Figure 1:**
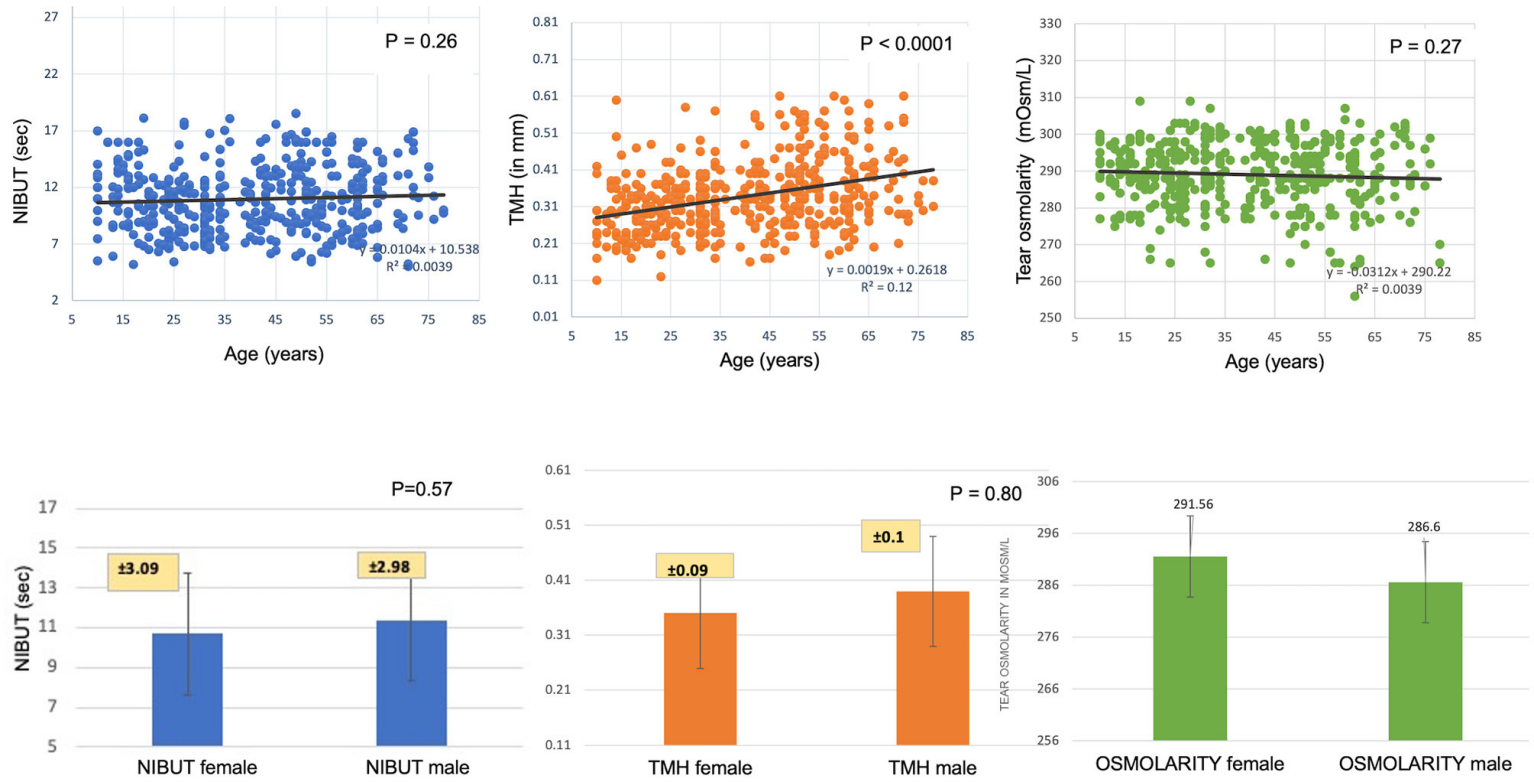
Age- and sex-wise distribution of tear meniscus height, non-invasive tear break up time, and tear osmolarity of 237 subjects.

A total of 276 participants took part in the investigation, out of which 237 fulfilled the OSDI criteria (<13). A total of 474 eyes of 237 healthy subjects, 150 males (63.29%) and 87 females (36.7%), were analyzed. The mean age was 40 ± 17 years (10-80 years). There were 9.7% (23/237) diabetics in the whole cohort, and 53% (126/237) had refractive errors (presbyopes, myopes). There were no differences noted in tear film parameters between emmetropes vs. individuals with refractive errors (*p* = 0.61). The participants were divided into six age-based groups (10-20, 21-30, 31-40, 41-50, 51-60, 61-80 years). The age-wise distribution of different parameters in both sexes is summarized in Table 1. Figure 1 shows the distribution of evaluated tear film parameters across different decades of life from the current study and the effect of age, sex on tear film parameters.

**Table 1:** Average values of evaluated tear film parameters across different age groups.

**Age, in years (M:F)**	**Mean TMH (in mm)**			**Mean NIBUT (in s)**			**Mean Tear osmolarity (mOsm/L)**		
	**Males**	**Females**	**Total**	**Males**	**Females**	**Total**	**Males**	**Females**	**Total**
10-20 (27:14)	0.29, 0.12	0.28, 0.06	0.29, 0.09	11.48, 2.93	11.81, 3.07	11.65, 3.00	289.27, 7.80	289.89, 8.50	289.58, 8.15
21-30 (30:10)	0.32, 0.07	0.30, 0.12	0.31, 0.098	10.11, 2.53	10.06, 2.65	10.08, 2.59	290.65, 8.47	288.9, 13.6	289.77, 11.05
31-40 (35:5)	0.30, 0.06	0.39, 0.10	0.35, 0.08	9.86, 2.48	10.95, 3.2	10.81, 2.84	289.76, 11.80	285.6, 4.94	287.67, 8.37
41-50 (28:12)	0.34, 0.10	0.40, 0.18	0.38, 0.14	11.44, 3.07	12.19, 3.08	11.81, 3.00	288.89, 7.22	288.37, 10.10	288.63, 8.67
51-60 (22:18)	0.42, 0.13	0.36, 0.10	0.39, 0.11	11.46, 3.02	10.50, 2.91	10.98, 2.97	286.93, 9.95	293.75, 9.63	290.34, 9.79
61-80 (18:18)	0.44, 0.13	0.36, 0.11	0.40, 0.12	11.70, 2.79	10.95, 2.99	11.33, 2.88	285.69, 9.98	289, 10.16	287.35, 10.07

*TMH, Tear meniscus height; mm, millimeters; NIBUT, Non-invasive tear break up time; mOsm/L, milliosmoles/Liter*.

### Tear Osmolarity

The mean tear film osmolarity of the healthy subjects was 289 ± 5.8 mOsm/L (range, 256-309). The tear osmolarity did not show any decade-wise variation (*P* = 0.27) or differences between males and females (*P* = 0.69). Also, tear osmolarity did not correlate with the TMH or NIBUT values (*P* = 0.83; *P* = 0.48). When considering the normal distribution of the mean value, 90% of the mean values were >270 mOsm/L.

### Tear Meniscus Height

The mean TMH of the healthy subjects was 0.34 ± 0.07 mm (range, 0.11-0.61). A positive binomial regression trendline was observed with age. Every 1-year increase in age led to an increase of 0.002 mm in the TMH value (*P* ≤ 0.0001). There were no differences between males and females (*P* = 0.83). The TMH values slightly negatively correlated with the NIBUT though they could not reach statistical significance (*P* = 0.69). The TMH values were independent of tear osmolarity.

### Non-invasive Tear Breakup Time

The mean NIBUT of the healthy subjects was 10.95 ± 2.02 s (range, 5.03-18.51). No differences were observed in NIBUT values with age (*P* = 0.26) or between males and females (*P* = 0.48). The NIBUT values had no correlation with the TMH (*P* = 0.12) or tear osmolarity values (*P* = 0.48).

## Discussion

Understanding physiological changes in tear film parameters in a normal population is significant in studying the pathophysiology of DED in the elderly population. The current study investigated the age and gender effect on tear film parameters measured in a non-invasive manner using Keratograph 5M. In the Indian population, TMH increases with age but does not correlate with NIBUT. NIBUT and tear film osmolarity show no significant age- or sex-related variation.

Many studies have proposed a reduction in tear production with age secondary to age-related atrophy of the lacrimal gland (5). Their conclusion uses invasive tests like the Schirmer test and fluorophotometry for estimating tear volume. Measuring Schirmer is different from the TMH, which is observed at the lower eyelid tear meniscus. TMH measured using OCT shows an age-related decline in normal individuals with a 1% decline per year (15, 16). We observed an increase in TMH values with increasing age, which was also reported by Patel et al. (17). The increase in TMH was noted more after 30 years of age in females and 50 years of age in males (Table 1). Although not statistically significant, higher average TMH values were noted in females compared to males in the 31-50-year age group. With age, the increase in TMH could be due to a reduction in the inferior forniceal volume or eyelid laxity, contributing to reduced tear outflow from the lacrimal system. We measured TMH values before NIBUT, which requires eyelid opening and induces reflex tearing (18). No gender-based variations were found in our cohort (17). Also, TMH values were unaffected by NIBUT readings. Golding et al. (19) reported a positive correlation between tear breakup time and TMH; poor TMH was associated with low tear breakup time values in DED subjects. It is in contrast to the findings of Patel et al. (17) where TMH values measured on tearscope did not relate to the lipid layer characteristics. Ideally, with a reduction in the tear volume, tear film becomes thinner and unstable. With age, the average NIBUT measurements did not show much change in normal subjects, and TMH values also increased; hence any effect of NIBUT on TMH is not expected.

Tear osmolarity values are considered a potential indicator of the severity of DED. Earlier, measuring tear osmolarity required laboratory osmometers, a large number of tear volumes, and a skilled technician, which has been made easy with the introduction of Tear lab handheld osmometers. The average reported normal tear osmolarity values are 300 ± 87.8 mOsm/L in a multicentric study measured in 314 subjects (20, 21). We found the mean osmolarity to be 289 ± 5.8 mOsm/L, measured in a non-invasive manner. Tear film evaporation rate has been shown to increase with age, which is reflected by elevated tear osmolarity values. Guillon et al. reported more values in older women than men, though measured using evaporimeter (9). No studies explored tear lab osmometer values over age and across gender. We did not find any relationship between age or gender and tear osmolarity values. Gender-based values have been reported using freezing-point depression nanolitre osmometry in one of the studies; no difference was found between males (306 mOsm/kg) and females (301 mOsm/kg) (4). In a study performed on 30 healthy subjects of Saudi origin, the mean tear osmolarity values were 299.066 ± 7.6 mOsm/L, which negatively correlated with TBUT values (mean NIBUT 12.1 s) (21). They had used the invasive technique of measuring TBUT; hence it cannot be compared with our study. In another study, tear osmolarity showed a very weak correlation with TMH values in healthy subjects but TMH was measured subjectively using Image J computation in their study (22). Our study did not find any correlation between TMH and tear osmolarity values.

Automated digital imaging software-based analysis of NIBUT makes the newly developed Keratograph more accurate and stable than conventional techniques. Keratograph measures NIBUT as the time taken for keratometry mires to become distorted after a complete blink. Though many studies have shown no gender-based differences in TBUT values, Craig et al. reported significantly lower NIBUT values (31.3 ± 25.4 vs. 23.8 ± 22.1) in females (4, 14, 23–28). Our study did not observe any differences in NIBUT values of men and women. Earlier studies have reported fluorescein TBUT values measured using slit-lamp biomicroscopy, which were reported to decrease with age (26). When keratograph was used for a population-based study in Chinese people, no age or gender-related differences were noted in NIBUT values (14). Similar to our study, no significant relationship was found between age and TBUT in Chinese, Indian, and African individuals (25). NIBUT has shown a good correlation with TMH measured using FD- OCT (14). We found no effect of NIBUT on TMH values, which could be due to large numbers of subjects being tested.

Most of the published studies have evaluated a single parameter, either TMH or NIBUT, in the normal population, restricted to a very defined age group, mainly middle-aged adults. The current study has the advantage of examining individuals from the second decade to the eighth decade of life in a large cohort with almost equal distribution among different age groups. We measured TMH values non-invasively under similar conditions and by the same observer, hence bias due to environmental conditions or different observers is less likely. The possible limitation of the study could be unequal male and female distribution in a few age groups, which reflects the hospital employee sex ratio. Also, the data is from hospital staff volunteers, hence may not be representative of normal Indian population.

In a normal Indian population, TMH is affected by age but is independent of sex, NIBUT, and tear osmolarity. Tear breakup time and osmolarity show no age- and no sex-related changes. The long thought notion that older individuals and women are at risk for DED is less likely to be related to changes in the tear film and could be due to other environmental or individual biological factors.

## Data Availability Statement

The data will be made available by the authors upon reasonable request.

## Ethics Statement

The study involving human participants were reviewed and approved by LV Prasad Eye Institute Ethics Committee. Written informed consent to participate in this study was provided by the participants' legal guardian/next of kin.

## Author Contributions

SSi, SSr, AM, and SB: concept and design of study or acquisition of data or analysis and interpretation of data and agreement to be accountable for all aspects of the work in ensuring that questions related to the accuracy or integrity of any part of the work are appropriately investigated and resolved. SSi, SSr, and SB: drafting the article or revising it critically for important intellectual content. All authors: final approval of the version to be published.

## Funding

This work was funded by Hyderabad Eye Research Foundation, Hyderabad, India.

## Conflict of Interest

The authors declare that the research was conducted in the absence of any commercial or financial relationships that could be construed as a potential conflict of interest.

## Publisher's Note

All claims expressed in this article are solely those of the authors and do not necessarily represent those of their affiliated organizations, or those of the publisher, the editors and the reviewers. Any product that may be evaluated in this article, or claim that may be made by its manufacturer, is not guaranteed or endorsed by the publisher.
